# Taxonomic Assignment-Based Genome Reconstruction from Apical Periodontal Metagenomes to Identify Antibiotic Resistance and Virulence Factors

**DOI:** 10.3390/life13010194

**Published:** 2023-01-09

**Authors:** K. Swapna Kumari, Sangita Dixit, Mahendra Gaur, Dibyajyoti Uttameswar Behera, Suchanda Dey, Rajesh Kumar Sahoo, Patitapaban Dash, Enketeswara Subudhi

**Affiliations:** 1 Institute of Dental Sciences, Siksha ‘O’ Anusandhan (Deemed to be University), Bhubaneswar 751003, India; 2 Centre for Biotechnology, School of Pharmaceutical Sciences, Siksha ‘O’ Anusandhan (Deemed to be University), Bhubaneswar 751003, India; 3 Drug Development and Analysis Laboratory, School of Pharmaceutical Sciences, Siksha ‘O’ Anusandhan (Deemed to be University), Bhubaneswar 751003, India; 4 Department of Biotechnology, Punjabi University, Patiala 147002, India

**Keywords:** apical periodontitis, root canal infections, metagenome, binning, diversity, multi-drug resistance, *Pseudomonas*, *Enterobacter*

## Abstract

Primary apical periodontitis occurs due to various insults to the dental pulp including microbial infections, physical and iatrogenic trauma, whereas inadequate elimination of intraradicular infection during root canal treatment may lead to secondary apical periodontitis. We explored the complex intra-radicular microbial communities and their functional potential through genome reconstruction. We applied shotgun metagenomic sequencing, binning and functional profiling to identify the significant contributors to infection at the acute and chronic apical periodontal lesions. Our analysis revealed the five classified clusters representing *Enterobacter*, *Enterococcus*, *Lacticaseibacillus*, *Pseudomonas*, *Streptococcus* and one unclassified cluster of contigs at the genus level. Of them, the major contributors were *Pseudomonas*, with 90.61% abundance in acute conditions, whereas *Enterobacter* followed by *Enterococcus* with 69.88% and 15.42% abundance, respectively, in chronic conditions. *Enterobacter* actively participated in antibiotic target alteration following multidrug efflux-mediated resistance mechanisms, predominant in the chronic stage. The prediction of pathways involved in the destruction of the supportive tissues of the tooth in *Enterobacter* and *Pseudomonas* support their crucial role in the manifestation of respective disease conditions. This study provides information about the differential composition of the microbiome in chronic and acute apical periodontitis. It takes a step to interpret the role of a single pathogen, solely or predominantly, in establishing endodontic infection types through genome reconstruction following high throughput metagenomic DNA analysis. The resistome prediction sheds a new light on the therapeutic treatment guidelines for endodontists. However, it needs further conclusive research to support this outcome using a larger number of samples with similar etiological conditions, but different demographic origin.

## 1. Introduction

Dental root canal infections are caused by microbial colonization within the pulp chamber and can penetrate the periradicular area through the apical and lateral foramina. A series of inflammatory reactions are triggered as the infecting microorganisms and their metabolites access the pulpal tissues and periradicular tissues around the infected tooth. These reactions induce proteolytic activity by various immune cells, e.g., polymorphonucleocytes, macrophages and mast cells, that results in the recruitment of osteoclasts at the site and the formation of a bony cavity [1]. Depending on the level of infection, the peri-radicular bony cavity thus formed is usually filled with cellular debris, microorganisms (both dead and alive), fibroblasts, osteoclasts, cholesterol crystals and immune cells in varying proportions [2,3].

The root canal infection process has a diverse etiology where a combination of multiple bacteria is considered to be the primary pathogens [4]. The initial invasion and colonization of the microorganisms into the pulp, causing necrosis and destruction of periapical tissues resulting in spontaneous pain, is diagnosed as acute apical periodontitis and is subjected to immediate endodontic intervention [5]. However, microorganisms cause chronic endodontic infections, either from acute infections that have resisted the insults from debridement procedures or when microorganisms enter the treated tooth and move into the periapical area through microleakage or coronal percolation [4]. In the dynamic process of disease progression, with the changes in environmental conditions, the microbiome adapts itself by altering its structure in terms of richness, evenness and diversity. There is a shift in prevalence and relative abundance of certain taxa to sustain the environmental changes and stresses for their survival and maintenance [6,7].

Despite that, the genomic characterization of these infecting bacteria and the bacterial members co-existing in the microbiome have been sparsely understood [7,8]. The microbial diversity of apical root canal infections has been investigated by cultivable and non-cultivable methods for years. The recent advancements in technologies for the molecular identification of microbiomes have revealed the presence and prevalence of many uncultivable bacterial species, thus expanding the catalogue of recognized and low-abundant endodontic microorganisms [9,10]. However, uncultivable method-based meta-analysis established the associations between the microbiome and human health conditions such as colorectal cancer [11,12,13] and obesity [12], including very few in root canal infections [8]. The Whole-genome Sequencing (WGS) method, introduced recently, has the potential for simultaneous evaluation and estimation of the existence and quantification of the microbiome potentially present in the oral samples collected from infected root canals [11,12,13]. Whole-metagenome shotgun sequencing provides detailed qualitative and quantitative information on all the classes of microorganisms, including viruses and eukaryotes, with strain-level data [14]. However, when combined with the clustering of meta reads/contigs and the extraction of high-quality genomes based on GC content, coverage and taxonomic classifications (binning) are very helpful in accommodating towards understanding the metabolic and functional contributions of individual microorganisms in a close environmental bacterial community. From an ecological perspective, detecting each member of a mixed conglomerate is crucial, as any member may act as a keystone species for the microbiome’s initiation, progression or establishment of virulence [15,16]. In this study, we focus on the bacterial composition and diversity analysis through the Illumina HiSeq Ten platform based on high throughput DNA sequencing to characterize the complex intra-radicular microbial communities in acute and chronic root canal infections.

Taking it one step further, in the present work, we constructed metagenome-assembled genomes (MAGs) by assembling sequence reads into scaffolds which in turn into candidate MAGs based on GC content, coverage, *k*-mer frequency patterns, complimentary marker genes and taxonomic alignments [8,17] as well as codon usage [8,18], followed by the taxonomic annotation and gene prediction. This method has the added advantage of exploring distinct functional properties and aids in understanding the complexity involved in human diseases [8,19,20] by overcoming the challenges associated with a high reliance on the limited information available on a smaller number of high-quality reference microbial genomes regarding specific etiological conditions [8,14,15]. Binning is the process of grouping reads or contigs and assigning them to individual genomes by reconstructing MAGs. Taxonomic binning corresponds to assigning a taxonomic identifier to sequence fragments based on information such as sequence similarity, sequence composition, or read coverage [21]. The robustness in binning comes from the grouping of contigs to assign them into the individual genome. This is carried out with reduced biasness of assigning the functional pathways and genes to specific genera, instead of relying grossly on short ORFs which may not bear hallmarks for protein-coding genes [22].

Further, inappropriate antibiotic recommendation habits of endodontists, even though they can be avoided for cases such as localized infection in uncompromised patients and irreversible pulpitis, lead to the emergence and spread of bacterial resistance [23]. Moreover, since the microbiome of the root canal serves as a reservoir for antibiotic resistance genes [23], it seems imperative to unravel antibiotic-resistant genes from the root canal.

The present study is undertaken with two major objectives of understanding the diversity and composition of predominant microorganisms differentially involved in two apical periodontal infection types—acute and chronic—using the uncultivable approach through the lowest common ancestor (LCA)-based genome reconstruction approach. We also explored the resistant and virulent determinants prevalent in their respective microbiomes.

## 2. Materials and Methods

### 2.1. Patient Selection

Of the patients diagnosed with apical periodontitis within the age group of 20–70 years, ten patients each with acute apical periodontitis and chronic apical periodontitis, respectively, were selected from the Out-patient Department of Conservative Dentistry and Endodontics, Institute of Dental Sciences, Siksha ‘O’ Anusandhan (Deemed to be University), Bhubaneswar, Odisha, based on their history, clinical symptoms and radiographic evaluation (Supplementary Table S1). Any single-rooted or multi-rooted teeth showing periapical radiolucency were included in the study.

The patients with the following criteria were excluded from the study: (i) patients who have received antibiotics within the last three months and suffering from any systemic disease; (ii) teeth with a curved root in the apical third portion of the root, those are difficult to isolate using a rubber dam and those with narrow canals that cannot be negotiated without solvents were excluded from the study; (iii) for chronic cases, those having any systemic disease or presence of any fractured endodontic file in any canal, as noticeable in intraoral radiographs, were excluded; and (iv) patients with endodontic treatment of the infected teeth performed within two years were also excluded.

### 2.2. Sampling

All samples were collected under the aseptic conditions mentioned in the previous studies [17,24]. After isolating the selected tooth with a pre-disinfected dam made up of rubber, the surrounding area of the tooth was cleaned using 30% hydrogen peroxide (H_2_O_2_), swabbed using 2.5% sodium hypochlorite solution (NaOCl), followed by inactivation of the area with 5% sodium thiosulfate (Na_2_S_2_O_3_). The cavity was prepared with a sterile bur at high speed. Following the previous routine, the teeth were sterilized again before entering the pulp chamber and then gained access to the root canal. Protaper Sx files were utilized first for coronal canal enlargement, followed by sterile K-files (08–15) to work up the peri-apical region of canals during primary treatments. The K-files were cut near the handle using a sterilized wire cutter and then transferred to a cryo-tube containing reduced transport fluid (RTF). Additionally, sterile paper points were introduced into the canal to the predetermined working length (using an electronic apex locator and pre-operative radiographs). Paper points were held for 1 min and then collected into the same cryotube containing RTF.

In retreatment cases, after gaining access to the root canal orifice, the gutta-percha from the coronal part of the canal was removed with Gates-Glidden drills and rest of the gutta percha was removed using hedstorm files. The recovered apical gutta-percha was collected in a sterile vial containing RTF during the instrumentation. The working length was established radiographically and also using an electronic apex locator. With minimal apparatus and no solvent, canals were widened consecutively until they reached a file size of 30’. Sterile paper points were introduced into the canal until the working length was held for one minute and transferred to the RTF medium’s vial. It was then transferred to the Centre for Biotechnology laboratory of our university within 30 min for further processing.

### 2.3. Metagenomic DNA Extraction, High-Throughput Sequencing and Bioinformatics Analysis

The total community DNA was extracted from the acute and chronic periodontal infection using the QIAamp DNA kit (Qiagen, Germany) according to the manufacturer’s instructions. The quality and quantity of the DNA were checked using 0.8% (*w/v*) agarose gel and Nanodrop™ (Thermo Scientific, Waltham, MA, USA), respectively. Ten samples of the same infectious conditions, each either acute or chronic, respectively, were pooled into one—not only to get high-quality and quantity DNA for sequencing for metagenomics analysis, but also to encourage minimum involvement of total labour, analysis cost and the implications associated with handling human samples [25]. Sample pooling is encouraged preferentially where there is the use of efficient high throughput genome analysis technology, such as the Illumina platform for next-generation sequencing, to reveal the microbial diversity when the less complex community is anticipated, and secondly when less difference in the prevalence of bacteria in similar epidemiological conditions is anticipated within a disease type studied (either acute or chronic root canal infection) [26]. The overall graphical representation of sample processing, from collection to DNA extraction, was presented in Supplementary Figure S1. The OD of absorbance value between 1.8 to 2.0 at 260/280 purity ratio and quantity of DNA ≥ 1 μg was considered as a quality standard for the construction of the DNA fragment library.

The DNA libraries were prepared using the Illumina NexteraXT DNA library preparation kit according to the manufacturer’s protocol. The MinElute PCR purification kit (Qiagen, Ltd., Crawley, UK) was used to clean up the fragmented DNA, following standard instructions. Prepared libraries were subjected to quantification of segmented DNA together with HyperLadder IV (Bioline, London, UK) to determine the approximate size of the DNA library. The libraries were then pooled in equimolar concentrations according to the Illumina standard protocol and sequenced at 2 × 150 bp on an Illumina HiSeq 2500 quick run, with duplicated samples across two lanes.

### 2.4. Quality Filtering, Co-Assembly and Extractions of MAGs

The raw reads were subjected to a quality check using FastQC v0.11.9 [27]. Based on the quality assessment of the raw reads, the end of the reads was trimmed and decontaminated by an Illumina adapter at a phred cutoff Q20. The trimming was performed using Cutadapt v1.8.1 [28]. The de novo co-assembly of the two pooled samples was conducted using MetaSPAdes v3.15.5 [29] with default parameters.

To cluster the contigs (length ≥ 1500 bp) and extract the MAGs, we used the Automata v2.1.0 pipeline [30]. Firstly, the quality-filtered reads were mapped to contigs to find their coverage, and then ORFs were predicted using Prodigal v2.6.3 [31] and then aligned to NCBI nonredundant (NR) protein database using Diamond v0.9.14.115 to identify their taxa. If contigs remain unclustered, they are recruited into clusters using the ML_recruitment.py script. The clustered contigs at the genus level were assigned as individual MAGs. The identified MAGs were evaluated using CheckM v1.2.2 [32] and BUSCO [33]. Those contigs clustered into genus/MAGs, having medium (≥20 to ≤90%) and high-quality (HQ) ≥90% genome completeness and ≤5% contamination, were selected for phylogenetic and comparative genomics analysis.

### 2.5. Phylogenetic and Functional Analysis

The boundaries of genetic relatedness of identified MAGs were evaluated using an orthologous fragment’s average nucleotide identities (ANIs) values via OrthoANI v3.7 [34]. The predicted MAGs were annotated using a rapid prokaryotic genome annotation pipeline (PROKKA v1.13) [35] with a default setting. To assign the molecular functions and pathways, we aligned the protein-coding gene sequences (CDS) of MAGs against the KEGG database using the kofamKOALA profile HMMs [36] with a cutoff e-value of 1 × 10^−5^. We further predicted the Clusters of Orthologous Groups (COGs) of CDS using the eggNOG-mapper tool [37]. Antibiotic resistance genes (ARGs) were classified by alignment against the Comprehensive Antibiotic Resistance Database (CARD) using RGI (v5.1.1) and AMRFinderPlus [38,39]. The virulent protein sequences were predicted using the Virulence Factor Database (VFDB) through BLAST (v2.10.1+) [40,41]. The single-copy and multi-copy orthologs and core proteomes across the MAGs were predicted by OrthoFinder v2.5.4 [42].

## 3. Results

### 3.1. Metagenomic Assembly of Apical Periodontal Microbiota

From the quality assessments of raw reads, we obtained 30.74 million high-quality reads from both the samples. The MetaSPAdes assembled these high-quality reads into 30,068 contigs ranging from 200 bp to 154,450 bp and 10,072 bp as N50. Only 4097 contigs had lengths ≥1000. All the contigs are larger than 1500 bp were subjected to an automated binning pipeline. Based on sequence homology, nucleotide composition, coverage, lowest common ancestor-based taxonomic assignment and the presence of single-copy marker genes, the automatic pipeline clustered these contigs into 31 genera.

### 3.2. Distribution of Contigs at Different Levels of the Taxonomy

**Figure 1:**
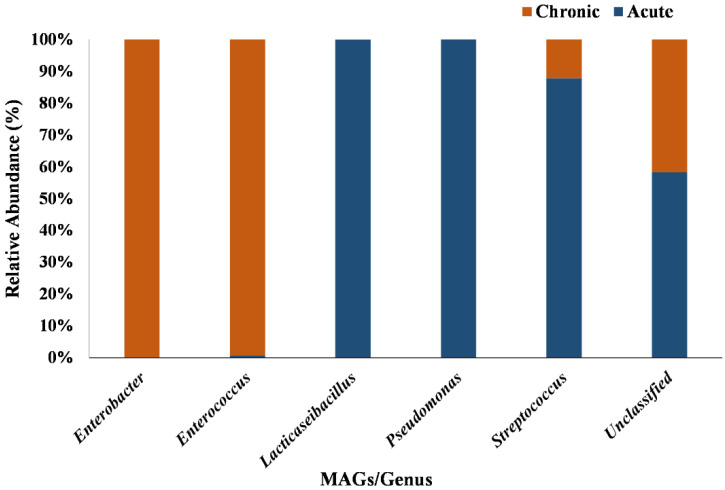
The distributions of relative abundance (%) represented as stacked bar plot for all MAGs accros the acute and chronic apical periodointitis. *Enterobacter* spp. and *Enterococcus* spp. are dominant in chronic conditions while *Lacticaseibacillus* spp., *Pseudomonas* spp. and *Streptococcus* spp. are dominant in acute conditions.

Five classified phyla and one unclassified phylum were recovered from acute and chronic samples. Of the major phyla, *Proteobacteria* (91.69%) was the most abundant in acute infections as compared to chronic infection (77.70%), while *Candidatus absconditabacteria* (0.046%), *Cyanobacteria* (0.10%), *Firmicutes* (18.79%) and unclassified (3.34%) were the most abundant in chronic infections (Supplementary Table S2). Overall, MAGs were assigned to seven different families, i.e., *Enterobacteriaceae*, *Enterococcaceae*, *Lactobacillaceae*, *Pseudomonadaceae*, *Spongiibacteraceae*, *Streptococcaceae* and unclassified. At the family level, out of the assigned seven families, *Pseudomonadaceae* were found to be the most abundant (91.03%) in acute conditions and *Enterobacteriaceae* were found to be most abundant (75.81%) in chronic conditions. The other most abundant family in the acute environment were *Lactobacillaceae* (3.86%) and *Streptococcaceae* (2.46%). While, *Enterococcaceae* (15.71%) and unclassified (6.88%) sp. were most abundantly present in chronic cases. The abundance of the *Spongiibacteraceae* (0.37%) family was uniquely found in acute cases (Supplementary Table S2). At the genus, a total of six MAGs (*Enterobacter*, *Enterococcus*, *Lacticaseibacillus*, *Pseudomonas*, *Streptococcus* and unclassified) were recovered. The abundance of *Pseudomonas* (90.90%), *Streptococcus* (2.45%) and *Lacticaseibacillus* (2.42%) were found to be highest in acute infection and the abundances of *Enterobacter* (69.15%), *Enterococcus* (15.71%) and unclassified (13.55%) were observed in chronic infection (Figure 1 and Supplementary Table S2).

From the taxonomic information, the corresponding abundances of the species derived from the two samples was assigned. A total of 35 species were classified in both samples. Out of that, 34 species were present in acute cases and 20 in chronic cases. There are 17 common species found in both acute and chronic conditions. The species *Pseudomonas helleri*, *Lacticaseibacillus rhamnosus*, *Pseudomonas* sp., *Pseudomonas marginalis*, *Pseudomonas fluorescens*, *Pseudomonas savastanoi* and *Spongiibacteraceae bacterium* were present most abundantly in the acute case whereas *Enterobacter cloacae*, *Enterococcus faecalis*, *Enterobacter roggenkampii*, *Enterobacter kobei*, *Enterobacter cancerogenus*, *Enterobacter* sp. fy-07 and *Streptococcus pneumoniae* were most abundantly present in chronic cases (Supplementary Table S2). Most of the species belonged to the *Pseudomonas* and *Enterobacter* genera both in acute and chronic infections. Species including *Pseudomonas marginalis*, *Pseudomonas fluorescens*, *Spongiibacteraceae bacterium*, *Pseudomonas aeruginosa*, *Pseudomonas putida* and *Pseudomonas paraversuta* were uniquely present in acute infections and the *Enterobacter mori* species was uniquely present in chronic infections.

### 3.3. Extraction of MAGs and Phylogenetic Analysis

**Figure 2:**
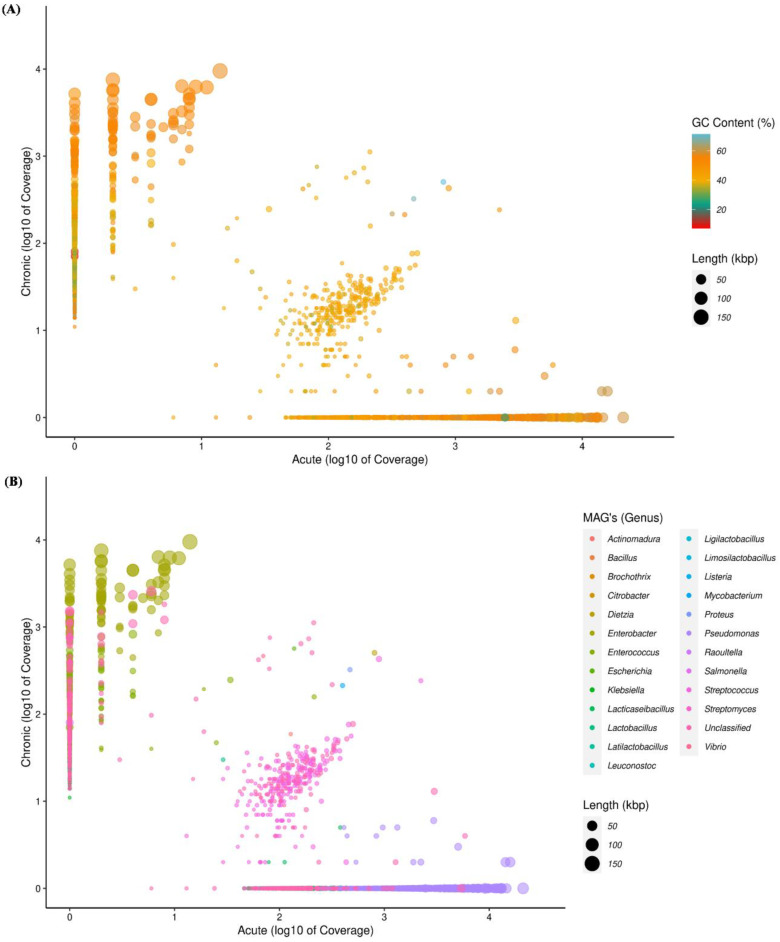
The scatter plot depicting the distributions of the contigs (≥1500 bp) and showing relationship between acute and chronic conditions. Each circle represents a contig and is colored by its GC content (**A**) and assigned taxonomy at the genus level (**B**). The length of contigs scaled the size of the circle in kbp.

The metagenomic taxonomy-based binning approach recovered 31 microbial MAGs at the genus level, with GC content varying from 31.25% to 71.25% (Figure 2A,B, Supplementary Table S2). Genera such as *Klebsiella*, *Citrobacter*, *Salmonella*, *Dietzia*, *Vibrio*, *Proteus*, *Plasmodium*, *Mycobacterium* and *Aspergillus* were dominant members of the microbiomes in chronic infection; and only *Pseudomonas* was dominantly found in acute infection (Supplementary Table S3). Within the highly scattered distribution (contigs) at the genus level, *Pseudomonas* was observed with the highest and *Rhizopus* was observed with the lowest scattered distribution (Figure 2B). Out of thirty-one MAGs, six MAGs were selected based on completeness and contamination of MAGs, according to MIMAG standards (>20% completeness and <5% contamination). A total of two MAGs were assigned to the “high-quality” (>90% completeness and <5% contamination), two MAGs to the “medium quality” (>50–90% completeness and <5% contamination) and two MAGs to the “low quality” >20–50% completeness and <5% contamination) and abundance. The six MAGs were assigned to four different phyla: *Proteobacteria* (MAG01, MAG04 and MAG06), *Firmicutes* (MAG02, MAG03, MAG05 and MAG06), *Candidatus Absconditabacteria* (MAG06) and *cyanobacteria* (MAG06). At the genus level, *Enterobacter* (MAG01), *Enterococcus* (MAG02), *Lacticaseibacillus* (MAG03), *Pseudomonas* (MAG04), *Streptococcus* (MAG05) and unclassified (MAG06) were assigned. Further, these six MAGs were assigned with thirty-six different species (Supplementary Table S2). The genome size of the six MAGs varied in length from 1.57 Mbp (MAG06) to 5.30 Mbp (MAG04) and their average %GC was 47.72 (Supplementary Table S3).

**Figure 3:**
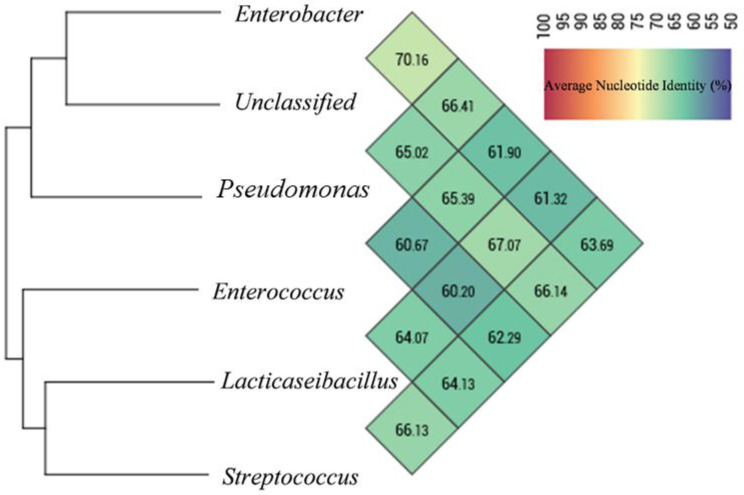
Hierarchical clustering of MAGs based on their average nucleotide identity (%; ANI). The high-quality MAGs extracted from draft co-assembly of acute and chronic metagenome sequencing data. The ANI is represented in upper half triangular matrix.

Clustering analysis through the tetraANI distance matrix showed MAGs were divided into two clades (Figure 3). The value of ANI between MAG01, MAG06 and MAG04, which are present in one clade, was 61.90 to 70.16% and the ANI threshold between the MAG02, MAG03 and MAG05 was between 60.20 to 66.29%. However, MAG03 shares low ANI (60.2%) with MAG04. The ANI measure in each MAGs ranged from 60.20 to 70.16%, strongly indicating that they are likely to be classified as new species of bacteria.

### 3.4. Functional Annotation of All the MAGs

**Figure 4:**
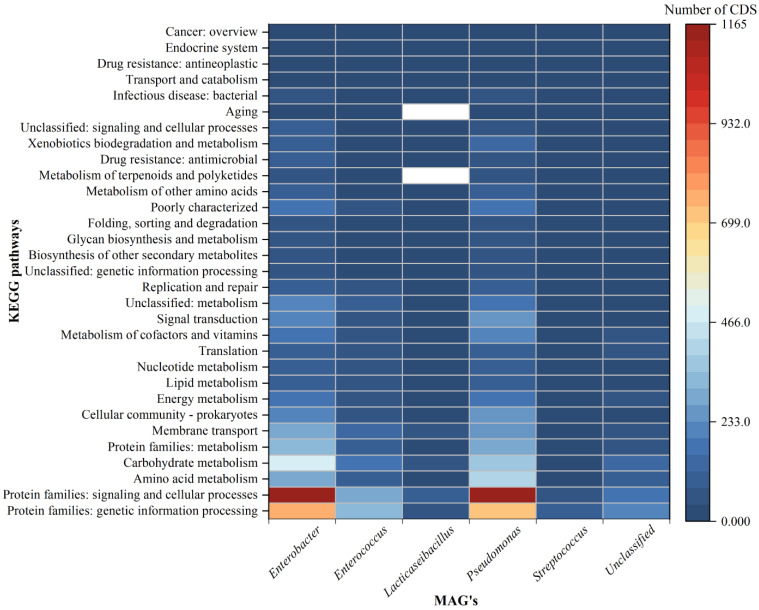
The heatmap illustrating the number of CDS and respective top twenty biological superpathways in extracted MAGs. The color bar on the right shows the CDS counts of individual KEGG orthologues.

Approximately 15,735 coding sequences (CDS) were predicted in all the MAGs tog "Clustering analysis through the tetraANI distance matrix ether. The lowest number of CDS was detected in the *Lacticaseibacillus* genus (MAG03) and the largest was in the *Enterobacter* genus (MAG01) (Supplementary Table S4). The number of ribosomal RNAs (rRNA) ranged from 1 to 5, transfer RNAs (tRNA) predicted in each MAG ranged from 4 to 66 and the transferred messenger RNAs (tmRNA) were not more than 1. The total number of sub-pathways found in all MAGs was 41 and the range of variation within the MAGs was very small. The most abundantly identified families, which were widely distributed across all the MAGs, were protein families (metabolism, genetic information, signaling and cellular processes), amino acid metabolism, carbohydrate metabolism, membrane transport, cellular community, energy metabolism, lipid metabolism, nucleotide metabolism and metabolism of cofactors and vitamins (Supplementary Table S5). The heatmap of the top 20 key pathways at the level 2 are shown in Figure 4, based on the gene count data. Most of the pathways are prevalent in *Enterobacter* and *Pseudomonas* followed by *Enterococcus* and unclassified bacteria, but least prevalent in *Lacticaseibacillus* and *Streptococcus*. The pathways responsible for membrane transport, antimicrobial resistance and infectious disease are found to be prevalent in *Enterobacter* and *Pseudomonas*, but least prevalent in *Streptococcus*. In a considerable number, the pathways related to cell growth and death, immune system, digestive system, endocrine and metabolic disease, cancer types, environmental adaptation and cell motility are detected in *Enterobacter* and *Pseudomonas* (Figure 4).

**Figure 5:**
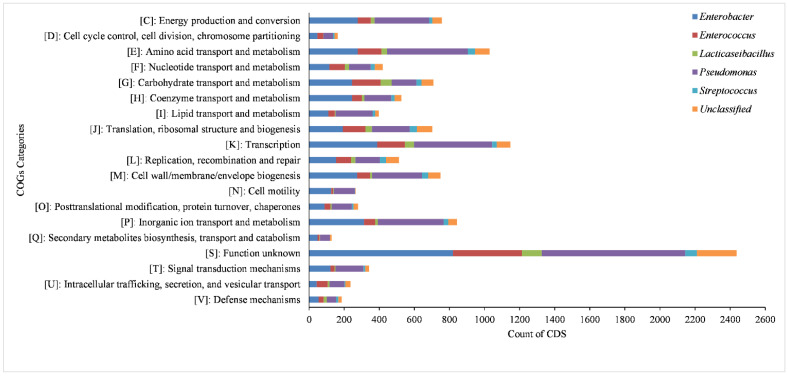
Horizontal stacked bar plot representing the counts of mapped CDS in Clusters of Orthologous Groups (COGs) functional categories in extracted MAGs.

The predicted coding sequences of the six MAGs yielded 11,832 distinct clusters of orthologous groups (COGs). The highest number of the COG category (4324) was found in *Pseudomonas*, followed by *Enterobacter* with 3944 and the lowest number of the COG category (420) was found in *Streptococcus* (Figure 5). A total of 19 COG categories are present in all the MAGs, out of which the highest category, found in the most abundant number—2439, has a function that is yet to be known. It is followed by amino acid transport and metabolism, inorganic ion transport and metabolism, energy production and conversion, cell wall/membrane/envelope biogenesis, carbohydrate transport and metabolism and translation, and ribosomal structure and biogenesis. Most of the above annotated pathways are prevalent in *Enterobacter* and *Pseudomonas* followed by *Enterococcus* and unclassified bacteria, but least prevalent in *Lacticaseibacillus* and *Streptococcus* (Figure 5).

### 3.5. Antibiotic Resistome of MAGs

To compare the ARG abundance between the acute and chronic groups, we calculated the number of ARG in each group based on the sequence coverage. The number of resistance genes in chronic was significantly higher than in acute (Table 1 and Table 2). A total of 32 types of ARGs were observed in both the samples. Among all the detected ARGs, 23 were found in *Enterobacter* (MAG01) in chronic infection and the most abundant types were β-lactam resistance, fluoroquinolone, glycopeptide, tetracycline, cephalosporin, phenicol, quinolone, monobactam, tigecycline and lincosamide. In the acute case, the most abundant ARGs were fluoroquinolone and cephalosporin found in *Pseudomonas* (MAG04). The resistance gene *ramA* was only found in *Enterobacter* and was most abundant in chronic, followed by *blaCMH-6*, *tet(34)*, *emrR*, *CRP* and *adeF*. The other ARGs were efflux pumps responsible for multi-drug resistance in chronic infection. The resistance gene *blaSRT* was only the most abundant found in an acute case of *Pseudomonas*, followed by *oqxB* and *TMex*C. Some AMR genes *tet*(M), *msr*(D) and *mef*(A) were only found in unclassified bacteria (MAG06) in chronic infection; however, genes such as *dfrE*, *lsa*(A) and *vanT* were detected in *Enterococcus* (MAG02) in chronic infection (Table 1 and Table 2). In both acute and chronic endodontic infections, most pathogens possess efflux-mediated antibiotic resistance genes, and more abundance was observed in chronic infection.

### 3.6. Virulome of MAGs

In total, 86 virulence factors (VFs) were recovered from all the MAGs in both cases. MAG04 was observed in the highest virulence factors (140 counts), followed by MAG01, MAG02, MAG06, MAG05 and MAG03 (Table 3). Offensive VFs were the dominant type in all the MAGs, with predominant subtypes including toxin, secretion system, and adherence (Table 3). When compared between chronic and acute endodontic infections, the abundance of virulence factors increased in the acute infection, mainly adherence, alginate regulation, iron uptake, immune evasion, secretion system and acid resistance. The chronic metagenomic sequence also revealed adherence, autotransporter, biofilm formation, fimbrial adherence, iron uptake and secretion system. Among the virulence factors, flagellar virulence factor (*flgABCDEFGHIJKLMN*, *flhAB*, *fliACDFGHIJKLMNOPQRST*, and *motABCD*) was the most abundant in an acute infection on *Pseudomonas*, followed by Type IV pili biosynthesis (*fimUV*, *pilCDEFGHIJNQRTVWX*), pyoverdine (*pvdADEGHIMOPQSY*), antiphagocytosis (*algPQRUVW* and *mucABDP*), Type IV pili twitching motility (*chpAC*, *cheV*) and *Acinetobactin* (*bauABCDE*). Similarly, SCI-I T6SS was the most abundant in a chronic infection on unclassified bacteria, followed by aerobactin siderophore (*iucABCD* and *iutA*) and Fimbrial adherence (*fimDFHIZ*, *stfD*, *stiB*, *stjBC*, and *stkBC*).

### 3.7. Orthologous and Core Proteome of MAGs

**Figure 6:**
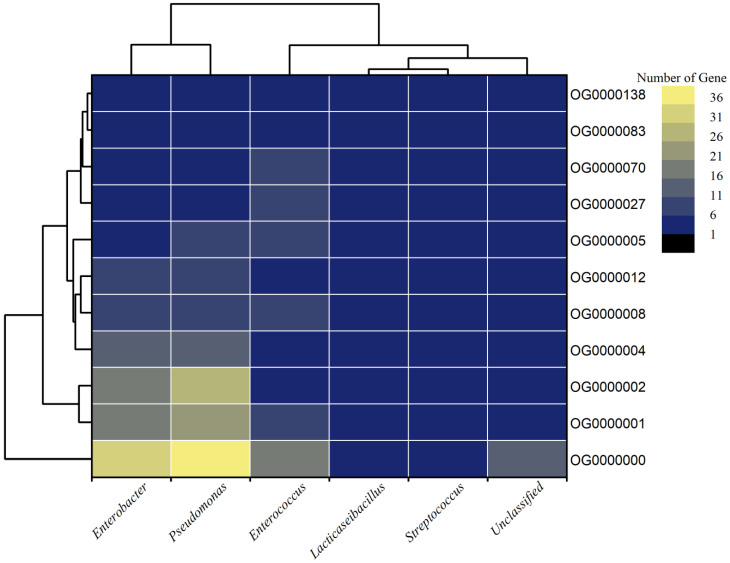
Heatmap with dendrogram of core orthogroups presents in all the MAGs. The heat color representing the number of genes assigned to each orthogroups in a particular MAG. The orthogroup OG0000000 shared the highest count (31–36) of genes from *Pseudomonas* spp. followed by *Enterobacter* spp. Similarly, most of the genes amongst all the orthogroups were shared by *Pseudomonas* spp. (3467 genes) and *Enterobacter* spp. (3159 genes; data not given).

To reconnoiter the orthologues and core functional proteomes of all the extracted high-quality MAGs, orthofinder was used to cluster the whole proteomes (13,734 genes) of all six MAGs. The whole proteomes clustered into 2690 orthogroups (OG’s; clusters of orthologous genes having a lowest common ancestor (LCA)) with a G50 (assigned genes) of 4, G50 (all genes) of 3 and O50 (assigned genes) of 709. A total of 9529 (69.4%) genes were assigned to 2690 orthogroups, whereas 4205 (30.6%) genes remained unassigned to any orthogroups. However, not a single-copy orthologue was predicted among the MAGs. Among all assigned genes, the highest numbers (3467) of genes in orthogroups were shared by *Pseudomonas* spp. followed by *Enterobacter* spp. (3159) (Figure 6). Similarly, 169 and 126 orthogroups were specifically present in *Pseudomonas* spp. and *Enterobacter* spp., respectively.

With the ≥95% cut-off value, only eleven orthogroups were present in all the six MAGs, belonging to different classes of functions. Three OG’s belong to the two-component regulatory system, two from each ABC-type ions/metabolites/amino-acid transport system and peptidoglycan/glycoside hydrolase. Whereas, one OG belongs to each class 1 translation termination release factor, efflux pump transcriptional activator, transcriptional regulator and Type I PRTase family (Supplementary Table S6). Orthogroup OG0000008 functions as an efflux pump transcriptional activator that regulates the expression of the AcrAB efflux pump under global stress conditions [43]. Orthogroup OG0000070, which belongs to the family of autolysins, catalyzes the hydrolysis of the β-1,4-glycosidic bond between the N-acetylglucosamine and N-acetylmuramic acid units of the bacterial peptidoglycan [44]. In doing so, they facilitate the genesis of daughter cells by cell division which might have a role in increasing the severity of the infection. Therefore, autolysins can be further explored as promising antibacterial therapeutic targets. Catabolite control proteins (OG0000005) are a major regulator of carbon metabolism in gram-positives and hence contribute to the severity of the infection [45]. The core orthogroups, which are differentially prevalent in acute and chronic apical periodontitis, can be a promising drug target to control the disease conditions in patients.

## 4. Discussion

A considerable amount of effort has been taken in depicting the microbiome of two different stages apical periodontitis infection to help in providing newer guidelines with the updated therapeutic regimen, and managing the prevalence of persistent endodontic infections. Our recent understanding from the characterization of the oral cavity microbiota, employing routine next-generation sequencing (NGS), establishes that these infections are associated with diverse microbiota with little differential abundance in few taxa in primary and secondary apical periodontitis infections. Having recorded dynamism existing in the microbial diversity and richness and the absence of a clear association of microbiota to different endodontic infection types, these study outcomes provide a non-specific composition but differential prevalence, per the clinical analysis [46]. So far, it has been understood that these types of infections are polymicrobial, and the severity of infection could be due to collective pathogenicity through the synergistic interaction of several bacterial species, contributing towards the community as a unit of pathogenicity. On the other hand, while searching for the major pathogen in endodontic infections, it is also speculated that the predominant species of any specific infection type must have played a critical role and might be responsible for establishing the virulence and pathogenicity of the group, instead of the population as a whole [47]. In the present work, we constructed metagenome-assembled genomes (MAGs) by assembling sequence reads based on taxonomic alignments, annotation and gene prediction. This method has the advantage of exploring distinct functional properties and has been used to understand the complexity involved in different human diseases [8]. It aids in overcoming the challenges associated with high reliance, primarily, on short ORFs, which may not bear a hallmark for protein-coding genes and secondarily, on the limited information available on a lesser number of high-quality reference microbial genomes about specific etiological conditions—more especially, the less understood root canal infections of the oral cavity [8]. The reconstruction of MAGs, from the binning through clustering of contigs that originated from the same source in the population, is used in the present study to compare with metagenomic sequences against the public database using alignment algorithms.

From the present binning analysis, we could reconstruct and finally recover only five classified MAGs; *Enterobacter* (MAG01), *Enterococcus* (MAG02), *Lacticaseibacillus* (MAG03), *Pseudomonas* (MAG04), *Streptococcus* (MAG05) and one unclassified (MAG06). Each one represents a genus with 36 species, out of 31 MAGs, from the total metagenomics sequence data which should be considered as incredible down streaming over the classical ORF based mg-RAST analysis that generates a huge number of genera. Based on the percentage coverage, we can assign the genus *Pseudomonas* with 90.90% of abundance in acute and the genus *Enterobacter* with 69.15% of abundance in chronic conditions of root canal infection (Figure 1). Though not highly conclusive, this could be one step forward towards establishing the role of a single major pathogen responsible for endodontic infection types (acute or chronic) over the concept of the polymicrobial nature of endodontic infections [47]. Overall, multiple deep sequencing from the same sample source/same etiological condition is a highly welcoming step to eliminate the bias acquired during sampling number and procedure. This can be done using tools and techniques for interpretation of results and establishing the decisive eco-physiological role of a single or most predominant bacteria in the definite root canal infection stage. High metabolic activity, higher infection and pathogenic ability are observed in MAG01 and MAG04, as predicted from the KEGG and COG analysis of all the MAGs (Figure 4 and Figure 5). This finding support the hypothesis that the two reconstructed genera *Pseudomonas* and *Enterobacter* are playing a crucial role in their respective acute and chronic infection environments, either singly or predominantly in a group.

In routine endodontic treatments, antibiotics are recommended for acute apical infections, or prophylactically for infections in medically compromised patients. Antibiotics have been proposed for some specific systemic or topical indications or used topically during “revascularization’’ procedures [48]. As of late, the oral microbiome of endodontic origin is also considered to be the reservoir of genes resistant to several classes of antibiotics, including tetracyclines, beta-lactams and macrolides [43,44,45]. Hence, realizing a pattern of prevailing antibiotic resistance in the endodontic microbiome will guide endodontists in selecting the most effective therapeutic regimens. A classical gold standard method of molecular detection of infecting bacteria and the prevailing resistance genes in the endodontic environments (acute or chronic) are still criticized for their inability to detect the difficult-to-grow or uncultivable bacteria from pus and root canal exudates from dentoalveolar infections [49,50].

In the present study, we detected the resistant genes from the metagenomics DNA of the root canal of acute and chronic infected teeth using the most advanced high throughput next-generation sequencing and following several bioinformatics tools. Among both the infection types, most of the species had antibiotic resistance mediated by efflux transporters. The *ram*A was responsible for the upregulation of the *Acr*AB efflux pump, which confers a multidrug-resistant phenotype to a variety of different antibiotic classes [51], whereas the *bla*CMH gene is responsible for cephalosporin (ceftazidime, cefotaxime and ceftriaxone) resistance [52]; *tet*(34) for tetracycline resistance; *fos*A for fosfomycin resistance; and *emr*R for nalidixic acid resistance. The oqxB and TMexC genes were responsible for resistance to fluoroquinolone and fourth-generation cephem [53]. *bla*SRT is a chromosomal novel beta-lactamase that confers resistance to third-generation cephalosporin antibiotics such as cefotaxime [54]. Rocas and Siqueira (2013) discovered the presence of *bla*TEM, *cfx*A, *erm*C, and tetM resistance genes in acute and chronic endodontic infections [23]; whereas, in this study, most of the resistance genes belonged to efflux-mediated resistance mechanisms, including RND efflux, major facilitator superfamily efflux pump, and ATP binding cassette efflux pump. These infections are distinguished by multispecies bacterial biofilms that regulate their internal environment by eliminating antibiotics through an efflux pump.

After analyzing our sequencing data, we identified various virulence factors which most likely contribute to the pathogenicity of infecting bacteria linked with biofilm development, immune evasion, inflammation and disease progression in both acute and chronic endodontic infections. The virulence genes *flg*, *flh*, *fli* and mot were responsible for the flagellar motility of the bacteria [55,56], whereas pil genes were responsible for surface and host cell adhesion, colonization, biofilm maturation, surface-associated motility and play horizontal genetic exchange [57]. It has been shown that pyoverdine (*pvdADEGHIMOPQSY*) functions as both a siderophore for scavenging iron (an essential nutrient) and a signaling molecule to produce virulence proteins during bacterial infections [58]. The antiphagocytosis virulence genes (*algPQRUVW*) encode alginate, which plays a crucial role in the production of biofilms [59]. Biofilms contribute to the persistence of bacteria in the oral cavity by acting as adhesins, keeping the bacteria from being evacuated and making it more challenging for phagocytes to kill the bacteria [60]. Type 1 fimbriae (*fimDFHIZ*) led to colonization and biofilm formation resulting in pathogenicity [61].

Similarly, T6SS enables the secretion of toxins and plays a role in interbacterial antagonism and biofilm formation [62]. Most aerobactins are found in pathogenic bacteria, but they also serve a crucial function in the transport of non-iron metals, defense against oxidative stress, antibiotic activity, interactions between species, and virulence [63]. The resistance gene profile, obtained from the above analysis, is found to be highly supported by the KEGG and COG-based prediction of pathways which are predominantly assigned as antimicrobial-resistant and most principally through efflux of drugs out of the bacterial cell in MAG01 and MAG04, followed by MAG02 and MAG06 (Figure 4 and Figure 5).

To complement the phylogenomic analysis for establishing demarcation among the reconstructed genus, genome-relatedness indices such as OrthoANI were implemented. This is a robust and faster means of measuring genome relatedness, which uses orthologous fragment pairs only [34]. Orthologous gene identification in MAGs showed more significant variations among all genera. Here, we proposed some newer members of bacteria from our study, which displayed shallow ANI values. Further, in our core proteome analysis, 11 core orthogoups were identified among all MAGs belonging to the two-component system, ATPase transporter, transcriptional regulator and autolysin, which can be targeted for antimicrobial drug and vaccine development for treating keystone crucial species that are predominantly responsible for specific endodontic infection types [64].

The present work is the first of its kind to interpret the differential microbial community involved in acute and chronic apical periodontitis through genome reconstruction using advanced bioinformatics tools and techniques. However, deep sequencing of a larger number of samples with the same etiological and varied demographic conditions is a highly welcoming step to eliminate the possible bias that might have been acquired during the present sampling procedure, before assigning a sole genus to an infection type, as well as to establish the decisive eco-physiological role in the definite root canal infection stage. In addition, integrated hybrid short-read and long-read sequencing and analysis techniques may be preferred over the present short-gun sequencing method alone to recover MAGs of better quality and quantity.

## 5. Conclusions

This study, for the first time, promoted a step to interpret the role of a single predominant pathogen in establishing endodontic infection types (acute or chronic) over the classical concept of polymicrobial infection through genome reconstruction from high throughput metagenomics DNA analysis, which needs further conclusive research in the line. It could predict the differential operation of antimicrobial resistance metabolic pathways, and could detect the efflux pump genes responsible for resistance to the most routinely prescribed classes of antibiotics in two infection types. This shall definitely shed new light on the preventive and therapeutic guidelines for endodontists regarding suitable treatment options through future research. The common core proteomes identified in this study can be targeted for the design and development of antimicrobial drugs and vaccines, in order to treat the crucial species that are predominantly responsible for specific endodontic infection types.

## Figures and Tables

**Table 1:** Occurrence of antimicrobial resistance (AMR) genes identified in the 6 MAGs with their relative abundance (%) in acute infection.

AMR Genes	Drug Class	Resistant Mechanism	*Enterobacter*	*Enterococcus*	*Lacticaseibacillus*	*Pseudomonas*	*Streptococcus*	Unclassified
*blaSRT*	Cephalosporin	Srt/sst family class c β-lactamase	-	-	-	0.033	-	-
*oqxB17*	Phenicol; Quinolone	Multidrug efflux RND transporter permease subunit oqxb17	-	-	-	0.042	-	-
*tmexC1*	Tetracycline	Multidrug efflux RND transporter periplasmic adaptor subunit tmexc1	-	-	-	0.029	-	-

**Table 2:** Occurrence of antimicrobial resistance (AMR) genes identified in the 6 MAGs with their relative abundance (%) in chronic infection.

AMR Genes	Drug Class	Resistant Mechanism	*Enterobacter*	*Enterococcus*	*Lacticaseibacillus*	*Pseudomonas*	*Streptococcus*	Unclassified
*adeF*	Fluoroquinolone; Tetracycline	Efflux mediated	0.119	-	-	-	-	-
*baeR*	Aminoglycoside; Aminocoumarin	Efflux mediated	0.011	-	-	-	-	-
*blaCMH-6*	β-Lactam	Class c β-lactamase cmh-6	0.016	-	-	-	-	-
*CRP*	Macrolide; Fluoroquinolone; Penam	Efflux mediated	0.013	-	-	-	-	-
*dfrE*	Diaminopyrimidine	Target replacement	-	0.003	-	-	-	-
*emrB*	Fluoroquinolone	Efflux mediated	0.009	-	-	-	-	-
*emrR*	Fluoroquinolone	Efflux mediated	0.012	-	-	-	-	-
*fosA*	Fosfomycin	Fosfomycin resistance glutathione transferase	0.011	-	-	-	-	-
*ftsI*	Cephalosporin; Cephamycin; Penam	Antibiotic target alteration	-	-	-	-	-	0.014
*H-NS*	Macrolide; Fluoroquinolone; Cephalosporin; Cephamycin; Penam; Tetracycline	Efflux mediated	0.007	-	-	-	-	-
*KpnE*	Macrolide; Aminoglycoside; Cephalosporin; Tetracycline; Peptide; Rifamycin; Disinfecting Agents and Antiseptics	Efflux mediated	0.007	-	-	-	-	-
*KpnF*	Macrolide; Aminoglycoside; Cephalosporin; Tetracycline; Peptide; Rifamycin; Disinfecting Agents and Antiseptics	Antibiotic efflux	0.008	-	-	-	-	-
*lsa*(A)	Lincosamide/Streptogramin	Abc-f type ribosomal protection protein lsa(a)	-	0.004	-	-	-	-
*marA*	Fluoroquinolone; Monobactam; Carbapenem; Cephalosporin; Glycylcycline; Cephamycin; Penam; Tetracycline; Rifamycin; Phenicol; Penem; Disinfecting Agents and Antiseptics	Antibiotic efflux; reduced permeability to antibiotic	0.006	-	-	-	-	-
*MarR*	Fluoroquinolone; Cephalosporin; Glycylcycline; Penam; Tetracycline; Rifamycin; Phenicol; Disinfecting Agents and Antiseptics	Target alteration; efflux mediated	0.004	-	-	-	-	-
*mef*(A)	Erythromycin	Macrolide efflux mfs transporter	-	-	-	-	-	0.007
*msbA*	Nitroimidazole	Efflux mediated	0.011	-	-	-	-	-
*msr*(D)	Erythromycin	Abc-f type ribosomal protection protein	-	-	-	-	-	0.002
*oqxA*	Phenicol/Quinolone	Multidrug efflux RND transporter periplasmic adaptor subunit	0.009	-	-	-	-	-
*oqxB*	Phenicol/Quinolone	Multidrug efflux RND transporter permease subunit	0.009	-	-	-	-	-
*patA*	Fluoroquinolone	Efflux mediated	-	-	-	-	0.002	-
*ramA*	Fluoroquinolone; Monobactam; Carbapenem; Cephalosporin; Glycylcycline; Cephamycin; Penam; Tetracycline; Rifamycin; Phenicol; Penem; Disinfecting Agents And Antiseptics	Efflux mediated; reduced permeability	0.02	-	-	-	-	-
*rsmA*	Fluoroquinolone; Diaminopyrimidine; Phenicol	Efflux mediated	0.006	-	-	-	-	-
*tet*(34)	Tetracycline	Oxytetracycline resistance phosphoribosyltransferase domain-containing protein	0.012	-	-	-	-	-
*tet*(M)	Tetracycline	Tetracycline resistance ribosomal protection protein	-	-	-	-	-	0.002
*uhpT*	Tetracycline	Target alteration	14.525	-	-	-	-	-
*vanG*	Tigecycline	Target alteration	0.011	-	-	-	-	-
*vanT*	Phosphonic Acid	Target alteration	-	0.008	-	-	-	-

**Table 3:** Summary of count of the identified virulence factors across 6 MAGs. The virulence factors identified using VFDB database.

VFclass	Virulence Factors	*Enterobacter*	*Enterococcus*	*Lacticaseibacillus*	*Pseudomonas*	*Streptococcus*	Unclassified
Acid resistance	Urease (Helicobacter)	-	-	-	2	-	-
Adherence	Acm	-	1	-	-	-	-
	AS	-	1	-	-	-	-
	Choline binding proteins	-	-	-	-	1	-
	Curli fibers	1	-	-	-	-	-
	*E. coli* common pilus (ECP)	2	-	-	-	-	-
	Ebp pill	-	4	-	-	-	-
	EcbA	-	1	-	-	-	-
	EfaA	-	1	-	-	-	-
	Flagella	-	-	-	44	-	-
	GroEL(*Clostridium*)	-	-	-	-	-	1
	Hemorrhagic *E. coli* pilus (HCP)	3	-	-	-	-	-
	LPS O-antigen (*P. aeruginosa*) (*Pseudomonas*)	1	-	-	1	-	-
	*P. fimbriae*	1	-	-	-	-	-
	Polar flagella (*Aeromonas*)	-	-	-	1	-	1
	Streptococcal plasmin receptor/GAPDH (*Streptococcus*)	-	-	-	-	-	1
	Type I fimbriae	3	-	-	-	-	-
	Type IV pili biosynthesis	-	-	-	13	-	-
	Type IV pili twitching motility related proteins	-	-	-	7	-	-
Alginate regulation	Alginate biosynthesis	-	-	-	2	-	-
	Antiphagocytosis	-	-	-	9	-	-
	Capsular polysaccharide (Vibrio)	1	-	-	3	-	-
	Capsule (Enterococcus)	-	-	-	-	1	-
	Capsule (Klebsiella)	2	-	-	-	-	-
Autotransporter	Contact-dependent inhibition CDI system	2	-	-	-	-	-
	EhaB	1	-	-	-	-	-
Biofilm formation	AdeFGH efflux pump/transport autoinducer	1	-	-	1	-	-
	BopD	-	1	-	-	-	-
	PNAG (Polysaccharide poly-N-acetylglucosamine) (Acinetobacter)	1	-	-	-	-	-
Efflux pump	AcrAB (Klebsiella)	-	-	-	3	-	-
Endotoxin	LOS (*Haemophilus*)	2	-	-	-	-	-
Enzyme	Gelatinase	-	1	-	-	-	-
	Hyaluronidase	-	1	-	-	-	-
	SprE	-	1	-	-	-	-
	Streptococcal enolase (*Streptococcus*)	-	-	-	-	-	1
Fimbrial adherence determinants	Fim (*Salmonella*)	5	-	-	-	-	-
	Stf (*Salmonella*)	1	-	-	1	-	-
	Sti (*Salmonella*)	1	-	-	-	-	-
	Stj (*Salmonella*)	2	-	-	-	-	-
	Stk (*Salmonella*)	2	-	-	-	-	-
Immune evasion	Capsule	-	-	-	1	-	-
	LPS glucosylation (*Shigella*)	-	-	-	1	-	-
	LPS(*Brucella*)	-	-	-	1	-	-
Invasion	Flagella (*Burkholderia*)	1	-	-	-	-	-
	(empty)	1	-	-	-	-	-
Iron uptake	Acinetobactin (*Acinetobacter*)	-	-	-	5	-	-
	Aerobactin siderophore	5	-	-	-	-	-
	Heme transport (*Shigella*)	1	-	-	-	-	-
	Heme uptake	3	-	-	-	-	-
	Periplasmic binding protein-dependent ABC transport systems (Vibrio)	-	-	-	1	-	-
	Pyochelin	-	-	-	2	-	-
	Pyochelin receptor	-	-	-	1	-	-
	Pyoverdine	-	-	-	12	-	-
	Pyoverdine receptors	-	-	-	1	-	-
	Pyoverdine (*Pseudomonas*)	1	-	-	-	-	-
	Salmochelin (*Klebsiella*)	-	-	-	1	-	-
Lipid and fatty acid metabolism	Isocitrate lyase (*Mycobacterium*)	-	-	-	1	-	-
	Pantothenate synthesis (*Mycobacterium*)	-	-	-	1	-	-
Magnesium uptake	Mg2+ transport (*Salmonella*)	-	-	-	1	-	-
Nutritional factor	Allantoin utilization (*Klebsiella*)	-	-	-	-	-	2
Others	O-antigen (*Yersinia*)	-	-	-	1	-	-
Protease	IgA1 protease	-	-	-	-	1	-
	Pla (*Yersinia*)	1	-	-	-	-	-
Quorum sensing	Acylhomoserine lactone synthase	-	-	-	1	-	-
Regulation	Carbon storage regulator A (*Legionella*)	-	-	-	1	-	-
	GacS/GacA two-component system	-	-	-	2	-	-
	Two-component system (*Acinetobacter*)	-	-	-	1	-	-
	Two-component system (*Bordetella*)	-	-	-	1	-	-
Secretion system	EPS type II secretion system (Vibrio)	1	-	-	1	-	-
	Flagella (cluster I) (Yersinia)	1	-	-	-	-	-
	Hcp secretion island-1 encoded type VI secretion system (H-T6SS) (*Pseudomonas*)	3	-	-	7	-	-
	Lsp type II secretion system (*Legionella*)	-	-	-	1	-	-
	*P. syringae* TTSS effectors	-	-	-	2	-	-
	SCI-I T6SS	7	-	-	-	-	6
	T2SS (Yst1) (*Yersinia*)	1	-	-	-	-	-
	T2SS (*Aeromonas*)	2	-	-	-	-	-
	T6SS-II (*Klebsiella*)	-	-	-	-	-	1
	T6SS-III (*Klebsiella*)	1	-	-	-	-	-
	T6SS (*Aeromonas*)	-	-	-	3	-	-
	TTSS (SPI-1 encode) (*Salmonella*)	1	-	-	-	-	-
Serum resistance	LPS rfb locus (*Klebsiella*)	1	-	-	-	-	-
Stress adaptation	Catalase (*Neisseria*)	-	-	-	1	-	-
	Manganese transport system (*Neisseria*)	1	-	-	1	-	-
Surface protein anchoring	Lipoprotein diacylglyceryl transferase (*Listeria*)	-	-	1	-	-	-
Toxin	Phytotoxin syringomycin (*Pseudomonas*)	1	-	-	-	-	-
	TccC-type insecticidal toxins	-	-	-	1	-	-

## Data Availability

The raw data used for the analysis in this study are submitted in NCBI’s BioSample repositories with the accession numbers SAMN21567195 (Acute) and SAMN21567196 (Chronic).

## Supplementary Materials

The following supporting information can be downloaded at: https://www.mdpi.com/article/10.3390/life13010194/s1, Figure S1: Graphical workflow of the sample collection and DNA extraction process implemented in this study; Table S1: The details of the patient from which samples were collected with their OPD case id, age, gender, resource teeth, symptoms and radiographic features; Table S2: Coverage, GC content, length (≥1500 bp), taxonomic classifications and relative abundance of contigs; Table S3: Qulitative assessment summary of the extracted MAGs at genus level with their relative abundance; Table S4: Summary of ORF predictions and annotation of MAGs; Table S5: Metabolic pathway recovered from 6 MAGs by the kofamscan result; Table S6: Identified core orthogroups (95–100%) with function of last common ancestor (LCA), description of conserved domain, function class and number of ORFs clustered in respective orthogroups.
