# Supplementary material for: Taxonomic Assignment-Based Genome Reconstruction from Apical Periodontal Metagenomes to Identify Antibiotic Resistance and Virulence Factors
Source: Life (Basel). 2023 Jan 9;13(1):194. doi: 10.3390/life13010194 (PMC9861942; doi:10.3390/life13010194)
Supplement: Supplementary file 1 [file life-13-00194-s001.zip › life-2097191-supplementary/supplementary_FigureS1.pdf]

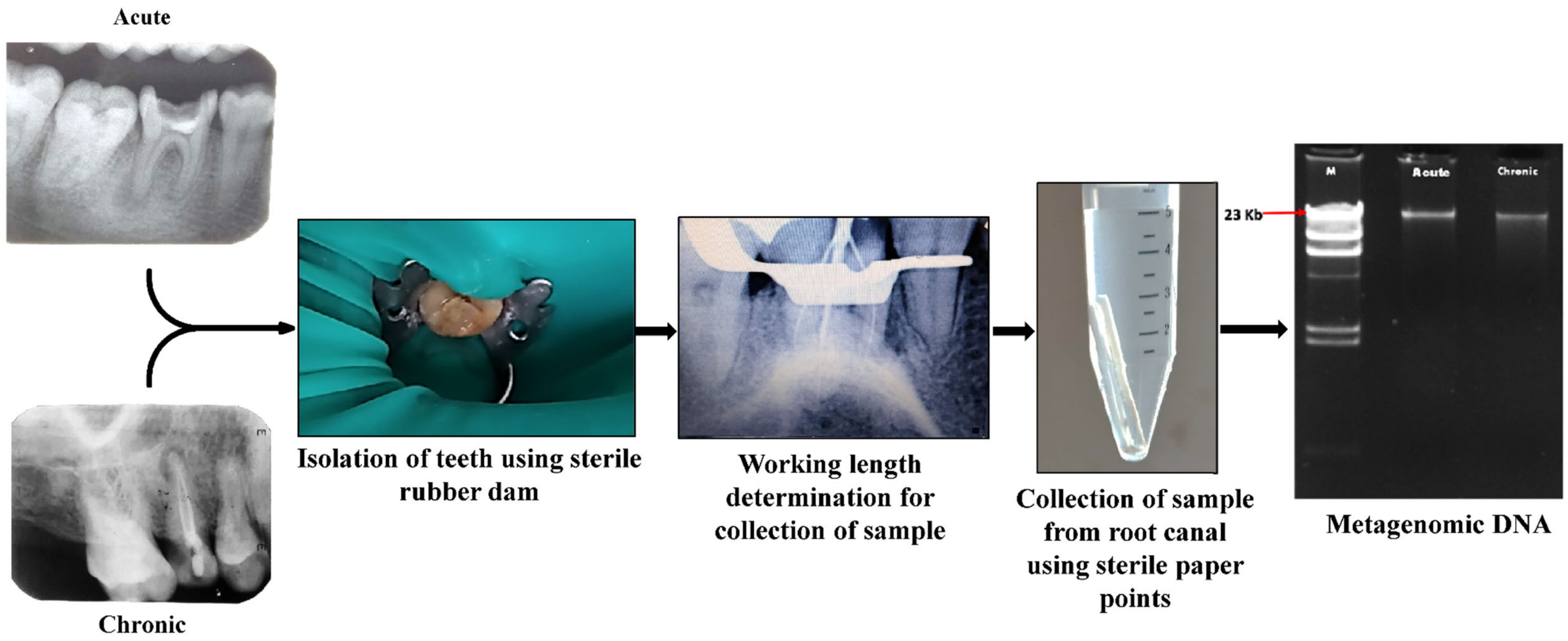

Supplementary Figure S1: Graphical workflow of the sample collection and DNA extraction process implemented in this study.
